# The Effectiveness and Safety of Acupuncture for Patients with Chronic Urticaria: A Systematic Review

**DOI:** 10.1155/2016/5191729

**Published:** 2016-05-25

**Authors:** Qin Yao, Shanshan Li, Xiaoxu Liu, Zongshi Qin, Zhishun Liu

**Affiliations:** ^1^Department of Acupuncture, Guang'anmen Hospital, China Academy of Chinese Medical Sciences, Beijing 100053, China; ^2^School of Graduates, Beijing University of Chinese Medicine, Beijing 100029, China

## Abstract

*Background*. Acupuncture might have effectiveness in relieving the symptoms of chronic urticaria. There are currently no systematic reviews of acupuncture for chronic urticaria published in English.* Objective*. We conducted a systematic review to assess the effectiveness and safety of acupuncture for chronic urticaria.* Methods*. A systematic review and meta-analysis of randomized, controlled trials were performed. The primary outcome was global symptom improvement.* Results*. We included 6 studies with 406 participants. Three trials showed significant difference between acupuncture and drugs in global symptom improvement (relative risk 1.37; 95% CI 1.11–1.70; *P* = 0.003). As an adjuvant to medication, acupuncture was also beneficial for global symptom improvement (relative risk 1.77; 95% CI 1.41–2.22; *P* < 0.01). There were no severe adverse events related to acupuncture.* Limitations*. Some methodological limitations were observed. The overall risk of bias in the 6 included trials was high and all included RCTs were conducted in China and published in Chinese. Besides, the lack of proper control groups and the use of different rating methods and cut-offs in the included trials also made the evidence of this review limited.* Conclusions*. Acupuncture might be effective and safe for chronic urticaria in relieving symptoms, based on a low level of evidence. To draw a reliable conclusion, more high quality trials are needed in the future. This trial is registered with PROSPERO CRD42015015702.

## 1. Introduction

Chronic urticaria is a common disease with recurrent pruritic wheals and/or angioedema continuing for more than 6 weeks [1–4]. The prevalence is 0.5–1%, and nearly 20% of people suffer from acute urticaria at least once during their lifetimes [5]. The peak age of incidence is between 20 and 40 years [5]. The incidence ratio is nearly 2 : 1 between women and men [5]. Chronic urticaria has a negative impact on the patient's quality of life as well as on the cost of social health care [2, 6, 7]. For the treatment, the first-line treatment of chronic urticaria is second-generation H_1_-antihistamines (sgAH) [1], which have minimal adverse events [8, 9]. If symptom persists, higher dosages (up to 4x) of sgAH will be given, followed by omalizumab (anti-IgE), cyclosporine A, or montelukast (leukotriene antagonist) [1].

Acupuncture has a long history and been widely used in clinical practice for treating chronic urticaria in China. Besides, studies signified acupuncture may be effective in relieving the symptoms of chronic urticaria [10–12]. Moreover, there are currently no systematic reviews of acupuncture for chronic urticaria published in English. The aim of this review is to evaluate systematically the effectiveness and safety of acupuncture therapy for patients with chronic urticaria. We expect that this review will provide more convincing evidence to help clinicians make decisions when treating chronic urticaria.

## 2. Methods and Analysis

The review protocol was registered on PROSPERO and was also published on BMJ Open [13]. The primary outcome was changed from improvement in pruritus and wheals in protocol to global symptom improvement in this review because the main outcome of the included RCTs was global symptom improvement.

### 2.1. Inclusion Criteria

We included randomized, controlled trials that evaluated acupuncture for patients with chronic urticaria [1–4, 14, 15]. The intervention comparisons consisted of acupuncture compared with no treatment/placebo/sham acupuncture [16]/other active therapies or acupuncture in addition to another active therapy compared with the same active therapy. Trials of acupuncture only compared with another form of acupuncture or a different type of traditional Chinese medicine (TCM) (e.g., Chinese herbal medicine) were excluded. The primary outcomes were global symptom improvement, measured by urticaria activity score (UAS) [2, 17] or other validated scales. The secondary outcomes included quality of life [18, 19] and the recurrence rate during the follow-up period. Adverse events were also assessed. Studies were only included when global symptom improvement was reported.

### 2.2. Literature Search

We electronically searched the following databases from their inception to January 2016: the Cochrane Central Register of Controlled Trials, PubMed, EMBASE, the Web of Science, Traditional Chinese Medicine databases, China National Knowledge Infrastructure, the Chinese Biomedical Literature Database, the Chinese Scientific Journal Database, and the Wan-Fang Database. We produced a search strategy based on the guidance of the Cochrane handbook guidelines [20]. The search strategy for PubMed is exhibited in Appendix. We also searched related conference proceedings, trial registers, and the reference lists of the identified publications for additional trials.

### 2.3. Data Collection and Analysis

We conducted the following processes in data collection and analysis: selection of studies, data extraction and management, assessment of risk of bias, dealing with missing data, assessment of heterogeneity, assessment of reporting biases, data synthesis, sensitivity analysis, subgroup analysis, and grading the quality of evidence.

Two of the review authors (Qin Yao and Zongshi Qin) independently screened the titles, abstracts, and keywords of the retrieved studies and further assessed the full texts. Excluded studies were recorded with explanations. Two authors independently extracted the data and completed the predefined data extraction form. General information, participants, methods, interventions, outcomes, results, adverse events, conflicts of interest, ethical approval, and other information were extracted. Disagreements were resolved by discussion between the two authors and arbitrated by a third author (Zhishun Liu) when necessary. Authors of the studies were contacted for clarification and missing data.

The risk of bias was assessed with Cochrane Collaboration's tool by two independent authors (Shanshan Li and Xiaoxu Liu), including the following domains: sequence generation, allocation sequence concealment, blinding of participants and personnel and outcome assessors, incomplete outcome data, selective outcome reporting, and other sources of bias. The assessments were classified into three levels: low risk, high risk, and unclear risk.

RevMan software, version 5.2, was applied for the data analysis and quantitative data synthesis. For dichotomous data, we used risk ratios (RRs) with 95% confidence intervals (CIs) for analysis. No continuous data were assessed in this review. A fixed effect model was used for data synthesis because all the *I* ^2^ test results were less than 50% in this meta-analysis. We decided not to detect reporting biases and small-study effects with funnel plots because of an insufficient number of studies. Subgroup analysis was not conducted for the same reason. Sensitivity analysis was not conducted because no significant heterogeneity was detected.

We used the Grading of Recommendations Assessment, Development, and Evaluation (GRADE) to judge the overall quality of the evidence for the primary outcomes. The following domains were assessed: risk of bias, consistency, directness, precision, publication bias, and additional points. The assessments were classified into four levels: high, moderate, low, or very low [21, 22].

## 3. Results

### 3.1. Study Selection and Study Characteristics

**Figure 1:**
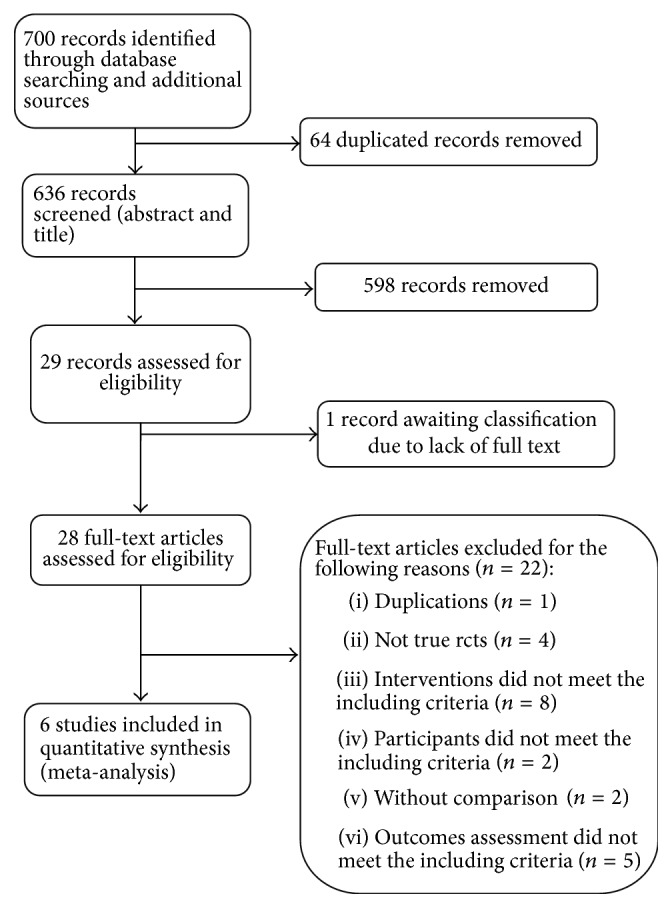
Study flow diagram.

We identified 700 references through electronic searches. In total, 6 RCTs with 406 enrolled participants were included (Figure 1) [23–28]. The characteristics of the included trials are summarized in Table 1. All included trials were conducted in China and were published in Chinese between 2005 and 2011 [23–25]. All included patients met the criteria for chronic urticaria. All included studies reported consistent baseline characteristics of sex and age.

In total, all the treatments adopted in the included trials were based on traditional Chinese medicine theory. Electroacupuncture was applied in 2 RCTs, while manual acupuncture was used in the other 4 RCTs. Xuehai (SP 10), Zusanli (ST 36), and Quchi (LI 11) were applied the most frequently in the included studies (4/6, 66.7%). Other acupoints used were Sanyinjiao (SP 6), Hegu (LI 4), Pishu (BL 20), Ganshu (BL 18), and Shendao (GV 11). The duration of the interventions was 2 weeks (2 trials) or 4 weeks (4 trials). The frequency of interventions ranged from once per day to twice per week. Only one trial mentioned a follow-up of 10 weeks. Three of the 6 RCTs compared acupuncture with pharmacological medications, and 3 of the 6 RCTs compared acupuncture plus medication versus medication. All drugs included belonged to the H_1_-antihistamine class.

All 6 trials reported global symptom improvement. For global symptom improvement, an effect index was employed for layering the strata in all the included trials, following a symptom score rating method: effect index = (baseline symptom score − symptom score after treatment)/baseline symptom score *∗* 100%. More than 60% was defined as remarkable improvement and the cure stratum in 5 of the included trials, and the remaining 1 trial used 80% as the cut-off point. We combined all the remarkable improvement and cure outcomes (more than 60%) into a single positive category and the remaining data into a negative category. Then, the new data were extracted as a dichotomous outcome for global symptom improvement. One trial reported the recurrence rate. Quality of life was not reported in any of the included trials. Safety was evaluated in all clinical trials.

### 3.2. Risk of Bias in Included RCTs

**Figure 2:**
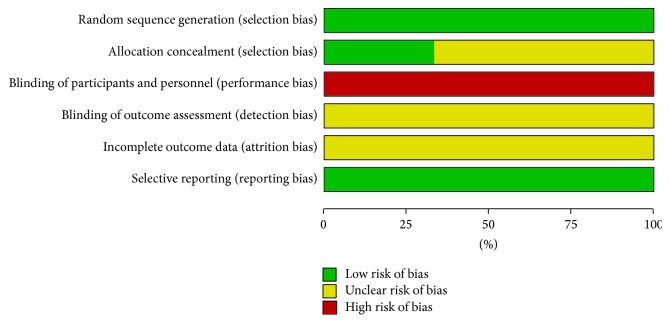
Chronic urticaria. risk of bias graph.

The risk of bias assessment is depicted in Figure 2. All included RCTs mentioned randomization. One RCT was randomized using random number tables, and it kept the allocation schedule safely concealed using opaque envelopes [23]. Another RCT was randomized using random number tables without mentioning whether the schedule is concealed or not [27]. The details of the randomization and allocation concealment were unclear in the remaining 4 RCTs, even after contacting the authors for advanced information. Moreover, none of the included RCTs blinded the acupuncturists, the participants, and the statisticians. Additionally, none of the included RCTs reported drop-outs, based on contacting of the authors or screening of the full texts.

### 3.3. Effect on Global Symptom Improvement

In this meta-analysis, 6 RCTs were divided into 2 parts to conduct the meta-analysis, depending on the different types of comparison groups.

#### 3.3.1. Acupuncture versus Medication

**Figure 3:**
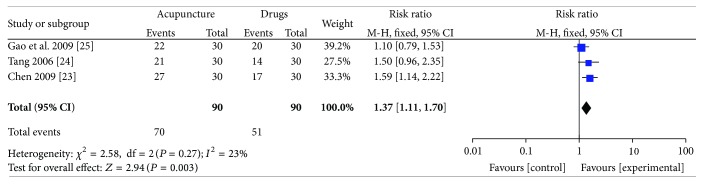
Forest plot of comparison: acupuncture versus medication; outcome: global symptom improvement.

Three trials [23–25] (180 participants) compared the effects of acupuncture versus medication (loratadine or cetirizine). There was a statistically significant difference between acupuncture and drugs in global symptom improvement. The RR for global symptom improvement was 1.37 (95% CI 1.11–1.70; *P* = 0.003; *I* ^2^ = 23%, Figure 3). GRADE analysis indicated that the overall quality of the evidence for this outcome was low due to a high risk of bias and the imprecision and sparseness of the data.

#### 3.3.2. Acupuncture Plus Medication versus Medication

**Figure 4:**
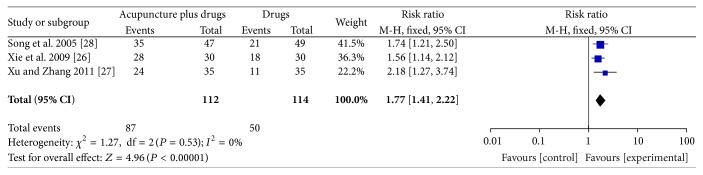
Forest plot of comparison: acupuncture plus medication versus medication; outcome: global symptom improvement.

Three studies [26–28] (226 participants) compared acupuncture plus medication versus the same medication with regard to global symptom improvement. The RR for global symptom improvement was 1.77 (95% CI 1.41–2.22; *P* < 0.01; *I* ^2^ = 0%, Figure 4). There was a statistically significant difference between acupuncture plus medication and medication regarding global symptom improvement. GRADE analysis indicated that the overall quality of the evidence for this outcome was low due to a high risk of bias and the imprecision and sparseness of the data.

### 3.4. The Recurrence Rate

The recurrence rate was evaluated by global symptom improvement. Only Gao's study [25] reported the recurrence rate. In Gao's report, acupuncture showed a lower recurrence rate than cetirizine over 10 weeks of follow-up (*P* = 0.06).

### 3.5. Quality of Life

None of the trials reported on quality of life.

### 3.6. Adverse Events

We included all clinical trials of acupuncture for treating chronic urticaria in a safety evaluation. There were only 15 articles out of the 212 of the clinical articles with 792 participants that mentioned safety evaluations related to acupuncture or acupuncture with other therapies to treat chronic urticaria. None of these studies reported any severe adverse events related to acupuncture. One trial reported 1 case of local skin hemorrhage and 1 case of feeling faint during acupuncture [29]. One trial reported 1 case of feeling faint during acupuncture [30]. Another trial reported 1 case of gastrointestinal discomfort, but the intervention in this trial was acupuncture versus Chinese herbal decoction [31]. One trial reported 1 case of menstrual disorders, but the symptoms disappeared after the intervention stopped, and the intervention in this trial was acupuncture versus cetirizine [32]. Moreover, a nervous feeling experienced by some patients when receiving acupuncture was reported [33]. None of the remaining clinical trials reported any adverse reactions related to acupuncture.

## 4. Discussion

The aim of this meta-analysis was to evaluate the effectiveness and safety of acupuncture therapy for chronic urticaria.

In total, 6 RCTs with 406 participants were included in this meta-analysis. The primary outcome was global symptom improvement. When comparing acupuncture with medications (loratadine, cetirizine), the combined results of 3 RCTs indicated that acupuncture might be more effective than drugs. As an adjunct to medication, the combined results of 3 RCTs suggested that acupuncture plus drugs was superior to drugs in improving global symptoms. The results indicated that acupuncture might have beneficial effects for the treatment of chronic urticaria as an adjunct to medication. However, the overall quality of the evidence assessed by the GRADE approach for this meta-analysis was low. There was a high overall risk of bias in the 6 included trials, which has also been found to be a common phenomenon in previous Chinese studies [34, 35].

First, the poor methodology of the included RCTs was a source of bias. The processes of randomization and allocation concealment were not clearly described in most of the included studies. No information was mentioned regarding the blinding of statisticians. None of these trials were registered in a clinical trials registry. Moreover, 2 RCTs were unpublished theses for Master's degrees [23, 24], and all included RCTs were conducted in China and published in Chinese, so the results should be explained cautiously.

The lack of proper control groups was another significant source of bias. Without comparisons of acupuncture to no treatment/placebo/sham acupuncture in this meta-analysis, we could not conclude that acupuncture had specific biological effects. All the included RCTs compared acupuncture to drugs or acupuncture plus drugs to drugs, and they did not conduct expectation evaluations. Thus, pretreatment preferences and greater expectations for acupuncture might have existed among the participants, and better responses might have been merged into the acupuncture group. Moreover, some articles implied that acupuncture might only have powerful placebo effects [36–38].

Regarding outcomes, although all the included RCTs reported global symptom improvement, different rating methods and cut-off points were applied. Because these rating methods and cut-off points were mostly self-defined, it is difficult to assess the effects of acupuncture by dichotomous outcomes. We chose only one cut-off point to combine all the positive outcomes into a single positive category and the remaining outcomes into a negative category, which might have resulted in differences between our results and those of the original studies. In addition, we hold the opinion that the effects of acupuncture might be better measured by a score rating method as continuous data than by being reprocessed into strata. Thus, we recommend that internationally acknowledged outcome measurements be applied and measured as continuous data, rather than strata, in future studies.

Only 1 of the included RCTs was designed to assess the patients' recurrence rate during the follow-up period. This RCT showed that acupuncture had a lower recurrence rate by 10-week follow-up, compared to cetirizine [25]. The results suggested that acupuncture might have better long-term effects than pharmacological therapy. Because clinically urticaria often occurs repeatedly, a longer follow-up is needed in future studies for an assured conclusion.

We conducted a descriptive analysis due to the variety of adverse reactions and the different types of clinical studies. So far, most of the studies have focused mainly on the effectiveness of acupuncture. We assessed 212 clinical articles related to acupuncture or acupuncture with other therapies to treat urticaria, of which only 4 trials with 5 cases reported tolerable and mild adverse reactions. None of the other trials mentioned any adverse reactions related to acupuncture. Therefore, we conclude that acupuncture is considered safe for treating patients with chronic urticaria.

## 5. Conclusion

In conclusion, there is low quality evidence for the effectiveness of acupuncture for chronic urticaria in relieving symptoms based on the results of this meta-analysis. Acupuncture is safe for treating patients with chronic urticaria, according to the current evidence. For future research, the quality of research must be improved, including the processes of randomization, blinding of outcome assessors, and longer follow-up. A consistent measurement for primary outcomes is necessary. The urticaria activity score (UAS) is recommended. Well-designed trials with larger sample sizes are necessary for reliable evidence regarding the effectiveness of acupuncture to treat chronic urticaria.

## Figures and Tables

**Table 1:** Chronic urticaria. The characteristics of included trials.

Reference	Comparisons	Methods	Course, wk	Outcomes
Chen 2009 [23] unpublished thesis	Electroacupuncture, 30; loratadine, 30	Random number table Adequate allocation concealment Blinding of outcome assessors: unclear	4	Global symptom improvement
Tang 2006 [24] unpublished thesis	Acupuncture, 30; loratadine, 30	Process of randomization: unclear Allocation concealment: unclear Blinding of outcome assessors: unclear	2	Global symptom improvement
Gao et al. 2009 [25]	Acupuncture, 30; cetirizine, 30	Process of randomization: unclear Allocation concealment: unclear Blinding of outcome assessors: unclear	2	Global symptom improvement recurrence rate
Xie et al. 2009 [26]	Electroacupuncture plus mizolastine, 30; mizolastine, 30	Simple random method Allocation concealment: unclear Blinding of outcome assessors: unclear	4	Global symptom improvement
Xu and Zhang 2011 [27]	Acupuncture plus cetirizine, 35; cetirizine, 35	Random number table Allocation concealment: unclear Blinding of outcome assessors: unclear	4	Global symptom improvement
Song et al. 2005 [28]	Acupuncture plus cetirizine, 47; cetirizine, 49	Process of randomization: unclear Allocation concealment: unclear Blinding of outcome assessors: unclear	4	Global symptom improvement

## Appendix

### A Search Strategy Used in PubMed

*(1) To Identify Relevant Outcomes (Chronic Urticaria)*. “Urticaria” [Mesh] OR Chronic urticaria [Title/Abstract] OR hives [Title/Abstract] OR nett-rash [Title/Abstract]) OR Angioedema [Title/Abstract] OR Fong-Tzen-Kwai [Title/Abstract] OR wind-rash-patch [Title/Abstract].

*(2) To Identify Relevant Exposures (Acupuncture)*. Acupuncture therapy [MeSH] OR Acupuncture [Title/Abstract] OR Acupoints [Title/Abstract] OR Acupuncture^*∗*^ [Title/Abstract] OR Body acupuncture [Title/Abstract] OR Scalp acupuncture [Title/Abstract] OR manual acupuncture [Title/Abstract] OR Auricular acupuncture [Title/Abstract] OR ear acupuncture [Title/Abstract] OR electroacupuncture [Title/Abstract] OR Fire needling [Title/Abstract] OR dermal needle [Title/Abstract] OR plum blossom needle [Title/Abstract] OR Pyonex [Title/Abstract] OR Abdominal acupuncture [Title/Abstract] OR Filiform steel needle [Title/Abstract].

*(3) To Limit to Epidemiologic Studies (RCT)*. Randomized controlled trial [Publication Type] OR Controlled clinical trial [Publication Type] OR Randomized [Title/Abstract] OR Randomly [Title/Abstract] OR Placebo [Title/Abstract] OR Trial [Title/Abstract] OR Groups [Title/Abstract].
